# Effect of the AHR Inhibitor CH223191 as an Adjunct Treatment for Mammarenavirus Infections

**DOI:** 10.3390/ijms27021071

**Published:** 2026-01-21

**Authors:** Miguel Angel Pelaez, Jonna B. Westover, Dionna Scharton, Cybele Carina García, Brian B. Gowen

**Affiliations:** 1 Laboratorio de Estrategias Antivirales, Departamento de Química Biológica, Facultad de Ciencias Exactas y Naturales, Universidad de Buenos Aires (UBA), Buenos Aires 1428, Argentina; miguelpelaez@qb.fcen.uba.ar; 2 Department of Animal, Dairy and Veterinary Sciences, Utah State University, Logan, UT 84322, USA; jonna.westover@usu.edu (J.B.W.); dionna.scharton@usu.edu (D.S.); 3 Instituto de Química Biológica de la Facultad de Ciencias Exactas y Naturales (IQUIBICEN), UBA-CONICET, Buenos Aires 1428, Argentina

**Keywords:** aryl hydrocarbon receptor, favipiravir, antivirals, viral hemorrhagic fevers, Junín virus, Tacaribe virus, synergy analysis

## Abstract

The family *Arenaviridae* encompasses zoonotic, rodent-borne pathogens (e.g., Lassa, Machupo, and Junín viruses) that cause severe viral hemorrhagic fevers with high case fatality rates. The current therapeutic landscape is severely limited, underscoring the urgent need for novel antiviral strategies. A promising approach involves combining directly acting antivirals with host-targeted antivirals. A compelling host-targeted antiviral target is the aryl hydrocarbon receptor (AHR). This ubiquitous ligand-activated transcription factor is a recognized pro-viral host factor across multiple viral families. Building on prior work with Junín and Tacaribe viruses, we investigated whether the AHR inhibitor CH223191 could enhance the virus-directed antiviral activity of favipiravir against these viruses. First, we evaluated the toxicity and antiviral potential of CH223191 against a lethal Junín virus infection in male and female hTfR1 mice. After demonstrating substantial protection, we conducted preliminary assays to study the antiviral effects of combining CH223191 and favipiravir on Tacaribe virus (TCRV) infections in the Vero cell culture model. We observed synergistic interaction with all four models (ZIP, Loewe, Bliss, and HSA). We next determined the sub-optimal dose of favipiravir and conducted an antiviral combination study in the AG129 mouse model infected with TCRV. The combination effectively protected mice from a lethal TCRV infection and showed cooperative effects, reducing weight loss and viral loads. Overall, these results show that the AHR is a promising pharmacological target for the development of novel antivirals. Furthermore, we discovered a cooperative interaction between the activities of favipiravir and CH223191.

## 1. Introduction

Viral hemorrhagic fevers (VHFs) are an increasing global health concern due to their potential to trigger outbreaks with high mortality rates [1,2]. VHFs encompass a wide range of illnesses, with clinical outcomes varying from mild to severe. Severe cases are typically marked by the abrupt onset of symptoms such as muscle and joint pain, fever, bleeding, and hypovolemic shock from blood loss. In the most severe cases, bleeding or hemorrhaging from bodily orifices and internal organs becomes one of the most noticeable symptoms [3]. Over the past two decades, several countries in the South American and Eastern Mediterranean regions have experienced significant outbreaks and sporadic cases of VHFs such as yellow fever and severe dengue fever [4,5,6] while the Eastern Mediterranean region has also faced increased incidences of Rift Valley fever and Crimean-Congo hemorrhagic fever [7,8]. Furthermore, the South American region has seen an alarming increase in Argentine, Brazilian, Bolivian, and Venezuelan hemorrhagic fevers caused by the Junín, Sabia, Machupo, and Guanarito mammarenaviruses, respectively [9,10,11].

The limited amount of effective antiviral drugs to combat VHFs makes the research and development of new and enhanced compounds a key aspect in global health. Among recent developments in the antiviral field, the compound T-705, or favipiravir (6-fluoro-3-hydroxy-2-pyrazinecarboxamide), has been characterized as a potent inhibitor of the viral polymerase [12]. Viral inhibition assays have shown that favipiravir affects the replication of a wide spectrum of viruses, including members of the *Orthomyxoviridae* (Influenza virus), *Bunyaviridae* (now part of the order Bunyaviricetes) (La Crosse, Punta Toro, Rift Valley fever, and sandfly fever viruses), and *Arenaviridae* (Junín, Pichinde, and Tacaribe viruses) [13,14]. An interesting clinical case in January 2020 involved a woman in Brussels diagnosed with Argentine hemorrhagic fever after having traveled to Argentina. The patient showed severe clinical manifestations, which led the clinicians to administer a combination treatment of ribavirin and favipiravir. After a 50-day hospitalization, the patient was discharged without sequelae [15]. While the favipiravir monotherapy continues to show promising results, it is not without obstacles.

A significant issue with direct-acting antiviral treatments against RNA viruses is the generation of resistant mutants. For example, a favipiravir-resistant mutant of Junín virus (JUNV) with two amino acid substitutions (namely the N462D substitution in the RNA-dependent RNA polymerase and the A168T substitution in the glycoprotein precursor) was isolated from the Candid#1 vaccine strain [16], exemplifying the capacity of RNA viruses to generate resistance to specific treatments. An attractive strategy to overcome the limitations of single drug treatments is the combination of compounds that block distinct, non-overlapping stages of the viral multiplication cycle [17,18,19]. Notably, combining drugs that target viral and host cell components offers several advantages. For example, combination drug treatment can minimize the impact of viral mutability on drug resistance. When host-targeted drugs are used in combination with direct-acting antivirals, viruses must develop resistance to two distinct mechanisms simultaneously, thereby reducing the likelihood of resistance emergence. Another advantage is the broad-spectrum potential of the combination treatment. Because several viruses hijack the same cellular processes for genome replication and viral assembly, targeting the host factors that drive these processes enables broad-spectrum antiviral effects.

In recent years, the aryl hydrocarbon receptor (AHR) has gathered attention as a potential host-directed therapeutic target against viral infections [20,21,22,23]. The AHR is a ligand-activated transcription factor expressed ubiquitously in animal cells. The primary role of this cytoplasmic receptor as a xenobiotic sensor was discovered in 1976 by Dr. Alan Poland’s laboratory at the University of Rochester, and elucidating its signaling pathway remains a promising research goal [24,25]. The AHR can sense ligands from various sources, including dietary sources, the gut microbiome, and anthropogenic pollutants. When activated, the canonical signaling pathway triggers the CYP family of monooxygenases and the metabolism of ligands. The AHR’s role in viral infections is still under investigation.

Our studies in the field show that inhibition of the AHR reduces viral replication across different cell lines, including those lacking innate viral suppression mechanisms [20,21]. Among the several AHR antagonist ligands, CH223191 (2-(1H-indole-3-carbonyl)-thiazole-4-carboxylic acid methyl ester) is usually chosen for its specific and potent inhibitory action. Beyond its emerging role in molecular virology, AHR inhibition serves as an important tool across diverse fields. For example, this inhibitory effect has been employed to modulate the AHR axis in cancer research to reverse disease progression [26], in immunology to evaluate AHR signaling in inflammatory skin diseases [27], and in toxicology to characterize the effects of fungicide exposure in human placental cells [28]. In this study, we sought to assess the combined potential of the AHR inhibitor CH223191 with the direct-acting antiviral favipiravir. We first evaluated the antiviral effectiveness of CH223191 *in vivo* using the hTfR1 mouse model challenged with JUNV. After observing promising results, an exploratory *in vitro* combination study was conducted in Vero cells to assess possible additive or synergistic effects. Subsequently, we performed *in vivo* studies to test the effectiveness of the combination treatments in a more tractable murine arenaviral infection model. Initially, the sub-optimal dose of favipiravir was determined to facilitate the resolution of potential synergistic effects. Finally, we conducted *in vivo* combination therapy in a second murine model of mammarenaviral disease to evaluate the protective and viral yield-reducing effects of both compounds, administered alone and in combination.

## 2. Results

### 2.1. Antiviral Effect of CH223191 and Favipiravir Combinations in Cell Culture

**Figure 1:**
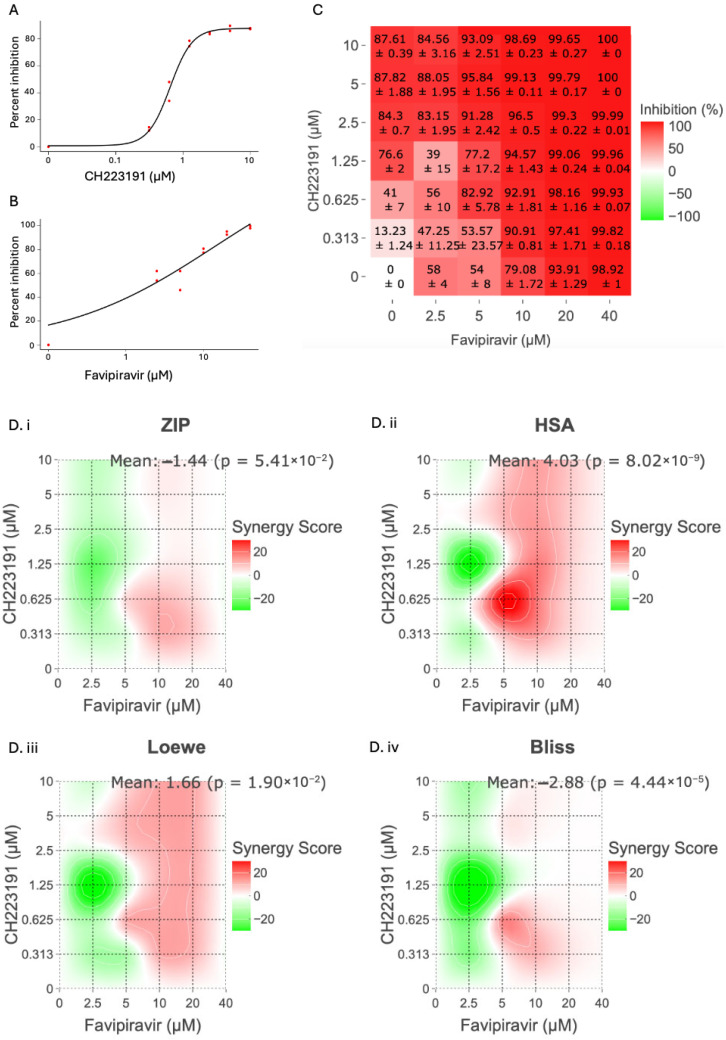
Determination of the *in vitro* antiviral effects of the CH223191 and favipiravir combination treatment. Vero cells were pre-treated with various combinations of CH223191 and favipiravir for 3 h. Afterwards, cell cultures were infected with TCRV for 1 h at 37 °C in 5% CO_2_. Following the infection, the inoculum was removed, and fresh drug-containing media were added to the cells. Cell supernatants were collected at 72 h p.i. Virus titer was determined by plaque-forming unit assay and the inhibition percentage was calculated relative to the untreated, infected control. (**A**) CH223191 dose response curve. (**B**) Favipiravir dose response curve. Red dots represent experimental replicates. (**C**) Inhibition matrix of CH223191 and favipiravir treatments. (**D**) Synergy analysis of the different drug combinations performed using 4 different synergy models (**D.i**) ZIP, (**D.ii**) HSA, (**D.iii**) Loewe, and (**D.iv**) Bliss. Synergy scores were calculated using the SynergyFinder web tool (https://synergyfinder.org). A score > 10 is considered synergistic, while a score < 10 and >−10 is considered additive, and a score < −10 is considered antagonistic. The data represent a compilation of the results from two experiments.

Given the growing evidence of CH223191’s antiviral activity across different experimental models [20,21,22,23], we decided to test the antiviral effect of combining the AHR antagonist with favipiravir. Favipiravir has already proven effective against several viral infections, including those caused by hemorrhagic fever viruses. To test whether a combination treatment with CH223191 could lower the effective dose of favipiravir, we conducted *in vitro* combination studies in Vero cells infected with TCRV (Figure 1). TCRV is a non-pathogenic, more tractable low-risk BSL-2 surrogate for the pathogenic New World mammarenaviruses, including JUNV.

As observed in previous studies, both compounds inhibited viral replication effectively as single-drug treatments in Vero cells infected with TCRV (Figure 1). Both compounds showed a dose–response effect (Figure 1A,B), with CH223191 achieving a maximum viral yield inhibition of 87.61% ± 0.55 [10 µM] and favipiravir an inhibition of 98.92% ± 1.41 [40 µM] (Figure 1C). The different combination treatments appeared to have distinct effects at different doses of either CH223191 or favipiravir (Figure 1C). Interestingly, we found that treatments between 5, 10, 20, and 40 µM of favipiravir, in combination with 0.313–10 µM of CH223191, showed a synergistic effect between both compounds (Figure 1D). In effect, all 4 models (ZIP, Loewe, Bliss, and HSA) indicated a synergistic effect between the 2 compounds, with single-point scores exceeding the 10-point threshold for synergy. Particularly, we observed peak scores of ZIP = 10.26 (CH223191: 0.313 µM; favipiravir: 10 µM) (Figure 1(D.i)), Loewe = 11.14 (CH223191: 0.313 µM; favipiravir: 5 µM) (Figure 1(D.ii)), Bliss = 14.81 (CH223191: 0.625 µM; favipiravir: 5 µM) (Figure 1(D.iii)), and HSA = 35.37 (CH223191: 0.625 µM; favipiravir: 5 µM) (Figure 1(D.iv)). However, the analysis also showed a strong antagonistic effect at CH223191 concentrations of 1.25 µM and favipiravir concentrations of 2.5 µM.

### 2.2. CH223191 Tolerance in the hTfR1 Mouse Model

**Figure 2:**
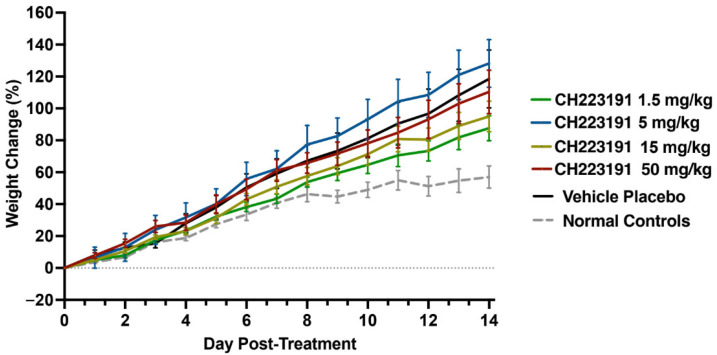
Maximum tolerated dose of CH223191 in hTfR1 mouse model. Animals in each group (*n* = 6/group) were administered 50, 15, 5, or 1.5 mg/kg CH223191 by PO route, q.d. for 10 days. A group of untreated mice (*n* = 4) was included for comparison. The mice were weighed daily and observed for 14 days for signs of toxicity. The weight data are represented as the group mean ± standard error of the mean (SEM) of the percent change in weight of animals relative to their starting weights on the day of treatment initiation.

The *in vitro* data obtained in the previous section (Section 4.1) support previous findings indicating that the AHR has a pro-viral role during *in vitro* infections with RNA viruses [22]. However, because these assays do not fully replicate the complexity of host immune responses, we sought to evaluate whether the observed activity translated to a physiological context. We first assessed the maximum tolerated dose of CH223191 in hTfR1 mice (Figure 2). All treated mice survived the 10-day treatment regimen and through the end of the study on day 14. The CH223191-treated mice had weight gain trajectories similar to those of the vehicle placebo control mice, with no signs of toxicity at any of the evaluated doses.

### 2.3. Evaluation of CH223191 in the hTfR1 Mouse JUNV Infection Model

**Figure 3:**
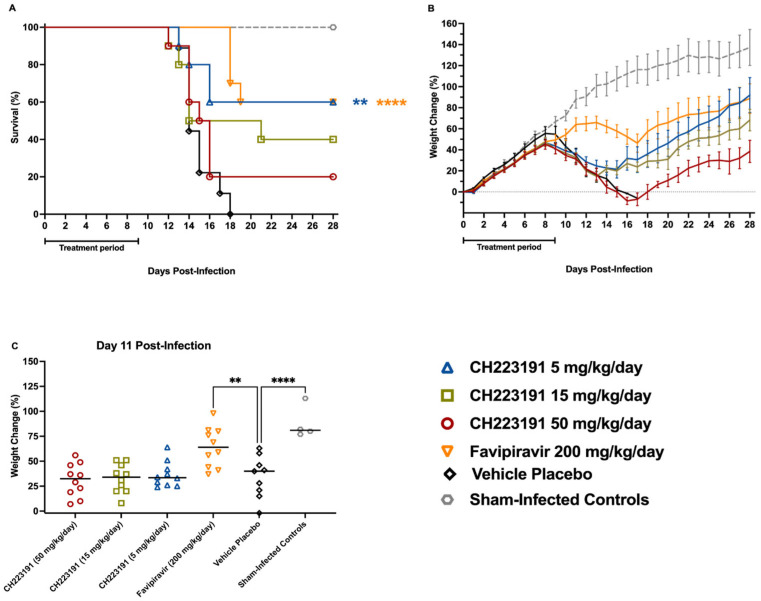
Effect of CH223191 treatment on survival outcome and body weight of hTfR1 mice challenged with JUNV. Animals in each group (*n* = 10/group) were treated PO starting 2 h pre-infection with a daily dose of 50, 15, or 5 mg/kg of CH223191. A treatment of 200 mg/kg of favipiravir was administered by IP injection to the positive control group. The animals were challenged by IP injection containing 10^4^ CCID50 of JUNV. Treatments were continued q.d. for 10 days. The animals were observed for 28 days to assess (**A**) morbidity and mortality and (**B**) daily body weights. Weight change data is represented as the group mean ± SEM of the percent change in weight of surviving animals relative to their starting weights on the day of virus challenge. (**C**) Percent weight change at day 11 p.i. Sham-infected animals were included as normal controls (*n* = 4). **** *p* < 0.0001, ** *p* < 0.01 compared to animals that received the CMC placebo.

After establishing that the dose range of 50–1.5 mg/kg/day of CH223191 was well tolerated with no detectable toxicity, we proceeded to assess whether the higher doses of CH223191 could confer protection against JUNV Romero infection in hTfR1 mice (Figure 3). The high and intermediate doses of CH223191 (50 and 15 mg/kg) provided low levels of protection with 20% and 40% survival, respectively. However, the 5 mg/kg dose of CH223191 significantly increased survival in mice compared to the untreated control (60% survival, ** *p* < 0.01), achieving the same survival rate as the favipiravir positive control (**** *p* < 0.0001) (Figure 3A). The CH223191 treatments appear to slightly delay the time at which animals succumb to infection compared to the placebo-treated control, although less than the favipiravir positive control. Notably, the beneficial effect of CH223191 on survival outcome followed an inverse dose response pattern.

Weight loss, a measure of disease severity in the JUNV mouse infection model, was consistent with the survival data (Figure 3B). The favipiravir-treated mice had the least amount of weight loss, followed by the middle- and low-dose CH223191-treated groups. The placebo-treated mice and high-dose CH223191 group had the most dramatic weight loss, with all the placebo-treated mice succumbing by day 18 p.i. All surviving mice were gaining weight at a similar trajectory at the study termination on day 28 p.i. Mice treated with the low dose of 5 mg/kg and the middle dose of 15 mg/kg of CH223191 had faster weight recovery than those treated with the 50 mg/kg dose, with the high-dose treatment closely tracking the placebo during days 0–16 p.i. The percent weight change for each animal on day 11 of infection shows a significant difference in weight between placebo-treated mice and the favipiravir group (Figure 3C).

### 2.4. Determination of the Favipiravir Sub-Optimal Dose in AG129 Mice Challenged with TCRV

**Figure 4:**
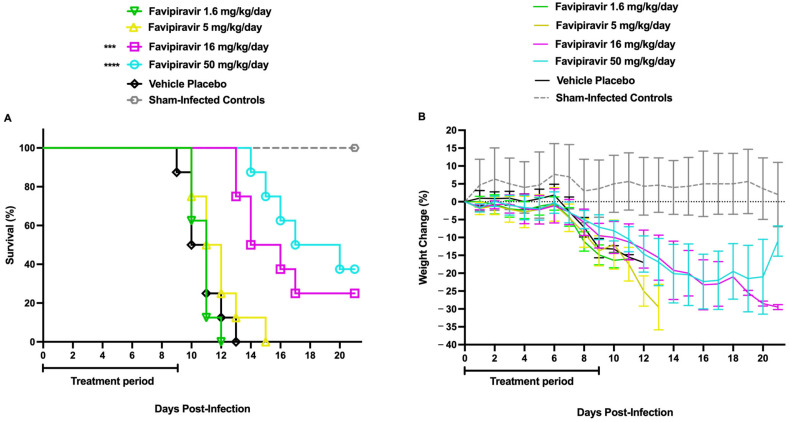
Determination of a sub-optimal favipiravir dose for TCRV infection in the AG129 mice. Animals in each group (*n* = 8/group) were treated PO starting 2 h pre-infection with 50, 16, 5, or 1.6 mg/kg/day of favipiravir. The animals were challenged IP with 500 CCID50 of TCRV. Treatment was continued b.i.d. for 10 days, and the animals were observed for 21 days for (**A**) mortality and (**B**) weight loss. Daily body weights are represented as the group mean ± SEM of the percent change in weight of surviving animals relative to their starting weights on the day of virus challenge. Sham-infected, normal control animals (*n* = 3) are shown for comparison. **** *p* < 0.0001, *** *p* < 0.001 compared to animals that received the CMC placebo.

To effectively study the combinatorial effect of both drugs in the established AG129 mouse model of TCRV infection, we first sought to identify a sub-optimal dose of favipiravir with reduced efficacy in protecting against mortality and weight loss (Figure 4). The daily 50 and 16 mg/kg doses of favipiravir modestly improved survival in mice infected with TCRV compared with placebo-treated controls (Figure 4A). These two treatments also delayed the onset of mortality (days 13–14) compared to the placebo and lower-dose treatment groups (days 9–10). Notably, the mice treated with the highest dose of favipiravir showed signs of weight recovery in the survivors (Figure 4B). Based on the data, a dose of 30 mg/kg/day was chosen so that both positive and negative effects of a combination could be resolved with the sub-optimal treatment.

### 2.5. Effect of CH223191 and Favipiravir Combination Treatment on TCRV-Infected AG129 Mice

**Figure 5:**
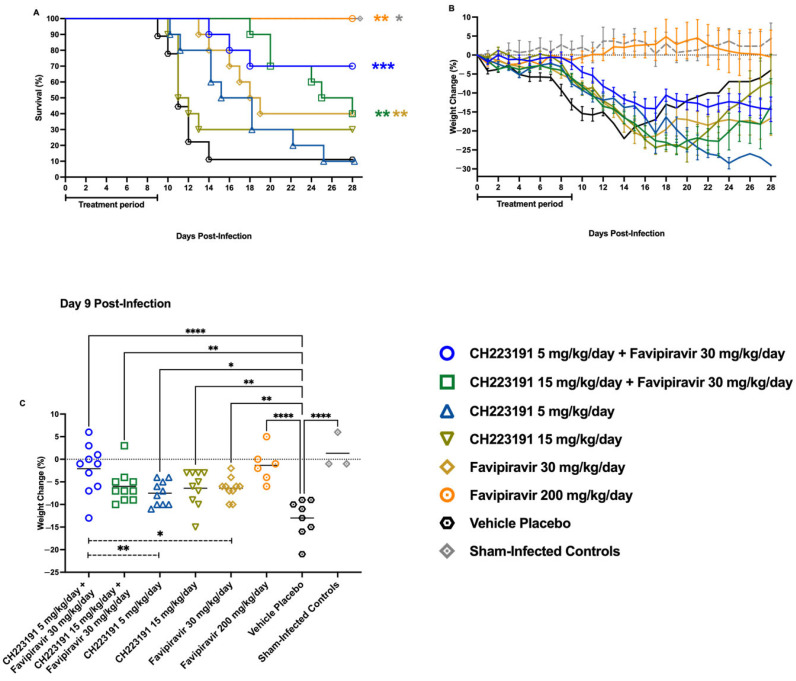
Effect of CH223191 and favipiravir combination treatments on survival outcome of AG129 mice challenged with TCRV. Animals in each group (*n* = 6–10/group) were treated PO starting 2 h pre-infection with two different dosing combinations of CH223191 and favipiravir. The animals were challenged with 500 CCID50 of TCRV. Treatments were continued b.i.d. for 10 days, and the animals were observed for 28 days for (**A**) mortality and (**B**) weight loss. Daily body weights are represented as the group mean ± SEM of the percent change in weight of surviving animals relative to their starting weights on the day of virus challenge. (**C**) Percent weight change at day 9 p.i. Sham-infected, normal control animals (*n* = 3) are shown for comparison. Dashed lines represent significant differences between the combination treatment of CH223191 (5 mg/kg/day) + favipiravir (30 mg/kg/day) and their respective monotherapies. **** *p* < 0.0001, *** *p* < 0.001, ** *p* < 0.01, * *p* < 0.05 compared to animals that received the carboxymethyl cellulose (CMC) vehicle placebo.

To assess the effectiveness of the CH223191 and favipiravir combination treatment against TCRV infection, the selected dose of 30 mg/kg/day favipiravir was combined with the doses of CH223191 (5 and 15 mg/kg/day) that performed the best in the hTfR1 mouse JUNV infection model. Consistent with previous results, the 30 mg/kg/day monotherapy protected only 40% of the infected mice, whereas the 200 mg/kg/day favipiravir positive control provided 100% protection (Figure 5A). Treatments with 5 or 15 mg/kg/day CH223191 did not significantly improve survival; however, the mice that received the lower dose and succumbed to the disease survived substantially longer (16 ± 4.9 **) than placebo-treated animals (11 ± 1.5; Table 1). When combined with the suboptimal favipiravir treatment, the 5 mg/kg/day dose of CH223191 provided significantly greater protection from mortality than the placebo group (*** *p* < 0.001, 95% CI of diff: −10.08 to 0.59) and greater protection than either CH223191 or favipiravir administered alone (Figure 5A). Notably, this combination was also significantly protective (* *p* < 0.05) by Fisher’s exact test (Table 1). Interestingly, when combined with suboptimal favipiravir, the higher 15 mg/kg/day dose of CH223191 extended the mean day to death (MDD) to beyond 3 weeks (23 ± 3.8 ****), but the number of survivors (40%) was no different than either drug treatment alone (30–40%; Table 1).

Individual weights recorded daily are consistent with the survival data (Figure 5B). Notably, the highest degree of protection based on body weight maintenance was observed with the suboptimal favipiravir (30 mg/kg/day) plus the low-dose 5 mg/kg/day CH223191 combination treatment group compared to the placebo-treated mice (**** *p* < 0.0001, 95% CI of diff: −15.82 to −5.98), and the average body weights were comparable to the positive control favipiravir treatment group (Figure 5C). However, we did not observe the expected weight recovery between days 18 and 28 that was observed in other groups. Moreover, we observed significant differences in the mean weights of mice treated with the low-dose 5 mg/kg/day CH223191 plus the 30 mg/kg/day favipiravir combination compared with those treated with their respective monotherapies.

**Figure 6:**
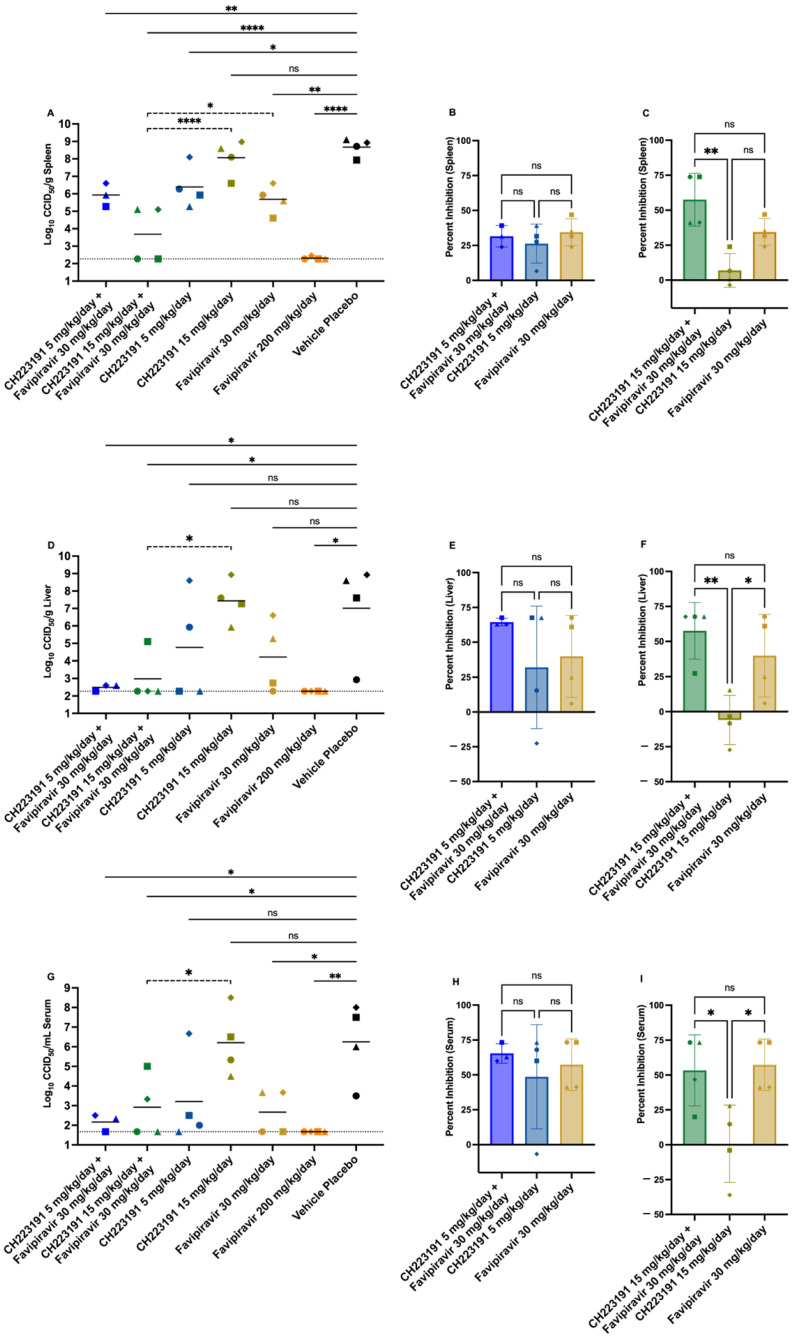
Effect of CH223191 and favipiravir combination treatments on viremia and tissue titers of AG129 mice challenged with TCRV. Animals in each group (*n* = 3–4/group) were treated PO starting 2 h pre-infection with two different dosing combinations of CH223191 and favipiravir. The animals were challenged with 500 CCID50 of TCRV. Treatments were continued b.i.d. for 10 days. Cohorts of mice were sacrificed on day 9 p.i. and spleen (**A**,**B**,**C**), liver (**D**,**E**,**F**), and serum (**G**,**H**,**I**) were analyzed for infectious virus. Colored symbols represent the same animals across all parameters. Horizontal lines represent the mean for each group. The gray dotted lines represent the lower limits of detection of the endpoint titration assays. Percent inhibition was calculated relative to the mean of the vehicle placebo and the error bars represent standard deviation. *ns* = not significant. **** *p* < 0.0001, ** *p* < 0.01, * *p* < 0.05.

In addition to the survival and body weight assessments, 4 mice from each group were preselected for viral titer analysis in spleen and liver tissues and viremia on day 9 p.i. In all cases, the CH223191 plus favipiravir combination treatments significantly reduced TCRV titers compared to the vehicle placebo (Figure 6A,D,G). The only treatment that did not significantly reduce viral loads in either tissue or serum was the 15 mg/kg/day dose of CH223191. Interestingly, the combination of 15 mg/kg/day of CH223191 with 30 mg/kg/day of favipiravir significantly reduced TCRV titer in the spleen compared with either treatment alone (Figure 6A). When analyzing the percent inhibition relative to the placebo-treated animals, we observed greater than an additive effect with the combination treatment (CH223191 15 mg/kg/day + favipiravir 30 mg/kg/day: 57.5%) compared to each individual treatment (CH223191 15 mg/kg/day: 6.95%; favipiravir 30 mg/kg/day: 34.5%) (Figure 6C), suggesting a cooperative effect between the compounds. Similar results were observed in liver samples (Figure 6E) and with viremia (Figure 6H), although the combination treatment did not significantly lower viral titers compared to the sub-optimal favipiravir (Figure 6D,G). The 5 mg/kg/day of CH223191 in combination with sub-optimal favipiravir showed a similar inhibitory trend (Figure 6B,F,I) compared to the 15 mg/kg/day dose of CH223191, with both combinations reaching inhibition percentages between 50% and 60% on the majority of the samples analyzed. However, no significant differences were observed between 5 mg/kg/day of CH223191 plus sub-optimal favipiravir and the corresponding monotherapies.

Collectively, the results show enhanced protection and antiviral activity when mice are treated with a sub-optimal dose of favipiravir administered in combination with the AHR antagonist CH223191.

## 3. Discussion

Amid a shortage of effective antivirals, the search for novel pharmacological targets is imperative. A promising tactic is combining compounds that target distinct aspects of the viral replication cycle. An example of this approach is the combined use of direct-acting and host-directed antivirals. This strategy of blocking distinct, non-overlapping stages of the viral replication cycle suppresses resistance development and increases the likelihood of broad-spectrum effects. Favipiravir is a well-studied, potent inhibitor of the viral RNA polymerase. Several studies have already evaluated the effectiveness of combination therapy between favipiravir and other compounds such as ribavirin [29], LHF-535 [18], lopinavir/ritonavir [30], and oseltamivir [17] to inhibit Lassa virus, JUNV, SARS-CoV-2, and Influenza H1N1 virus, respectively. Building on this line of research, growing attention has been directed toward host cellular pathways that may influence viral replication or therapeutic response. In recent years, the AHR has emerged as a key regulator of developmental, physiological, and disease-related pathogenic processes [31,32]. In particular, the AHR’s role in modulating the transcription of key genes involved in the antiviral response and the viral replication cycle makes this cytoplasmic receptor a promising target for the development of host-directed therapy. While there are currently no clinical trials evaluating the safety and efficacy of the AHR inhibitors for the treatment of viral infections, recent trials tested the AHR inhibitors IK-175 [33] and BAY 2416964 [34] for the treatment of solid tumors. Our results show that CH223191 is a potent AHR antagonist with proven antiviral activity across several viral infection models.

In this study, we expanded our investigation to include *in vivo* experiments using established mouse models of mammarenaviral disease. This is the first report demonstrating significant protection against viral infections in mice afforded by treatment with an AHR antagonist. These results align with *in vitro* data from previous studies [22], further supporting the AHR pathway as a promising pharmacological target for treating viral infections. Surprisingly, we observed that higher doses of CH223191 were less effective (Figure 3A). While the range of administered doses (5–50 mg/kg/day) was determined to be non-toxic in uninfected hTfR1 mice (Figure 2), the stresses caused by both CH223191 treatment and JUNV infection presumably contributed to the reduced efficacy. A possible biological explanation can be found on the crosstalk between NF-κB and the AHR. The effect of infectious diseases on drug metabolism and pharmacokinetics has been thoroughly studied [35]. The NF-κB transcription factor is a key regulator of inflammation that is particularly active during viral infections [36] and has been implicated in the regulation of cytochrome P450 enzymes, which mediate the metabolism of CH223191, via interaction with their induction mechanisms. NF-κB binds to the AHR, and these two transcription factors can repress each other’s function [37]. This interaction, in addition to CH223191’s inhibition of AHR, could prolong CH223191’s half-life and increase its toxicity in infected mice. However, the available evidence does not support this as the only possible explanation. For example, another plausible explanation could be that over-inhibition of the AHR causes an exacerbated inflammatory response as observed in skin [38] and gut tissue [39,40]. This effect leads to higher mice mortality when treated with higher concentrations of CH223191. The wide array of biological processes that the AHR has effect over makes this an interesting subject for further mechanistic studies.

More importantly, we observed that a dose of 5 mg/kg/day significantly protected infected hTfR1 mice from JUNV infection. While this protection was comparable to that of the 200 mg/kg/day favipiravir positive control, the dosing frequency of favipiravir treatment was not optimal, likely reducing its efficacy. Notably, we did not observe the same levels of protection when treating AG129 mice with 5 mg/kg/day of CH223191 alone (Figure 5). It is possible that the effect of AHR inhibition on viral pathogenesis was greater in hTfR1 mice than in AG129 animals, because the former possesses an active, competent type I IFN signaling pathway. The AHR can modulate the expression of a wide array of immune response genes through NF-κB signaling, as previously mentioned. Some of these genes encode different types of IFNs, key molecules involved in the antiviral response. Studies have shown that knocking out the AHR leads to a greater expression of type 1 IFNs *in vitro* [41] and of IFN-γ *in vivo* [42]. Furthermore, inhibition of the AHR with a specific antagonist, such as CH223191, promoted differentiation to IFN-gamma producing NK cells [43]. The marked difference in the effectiveness of CH223191 can be attributed to the absence of active IFN-α/β and γ signaling pathways, which can be indirectly enhanced by AHR inhibition in AG129 mice. It is important to note that while the absence of this signaling pathway in AG129 mice limits their representation of an immunocompetent state, they nonetheless establish that AHR inhibition provides moderate protection against TCRV infection. This effect, occurring independently of IFN signaling, broadens the current understanding of how AHR modulation influences viral outcomes.

Previous results show that AHR inhibition can affect viral replication even in the absence of active IFN-α/β and γ signaling [21]. The results of the *in vitro* combination assay showed that this is also the case for TCRV infections (Figure 1). Both CH223191 and favipiravir effectively inhibited TCRV replication, alone and in combination, corroborating previous findings (Figure 1B). Interestingly, synergy analysis showed a relatively high single-point score on all four models evaluated (Figure 1D). It is important to note that for the CH223191 and favipiravir combination, the most accurate models for synergy prediction are ZIP (Figure 1(D.i)) and Bliss (Figure 1(D.iii)). This is mainly because they align most closely with the known pharmacological profiles of CH223191 and favipiravir. CH223191 modulates host AHR signaling, a pathway that influences the cellular environment required for efficient viral replication, whereas favipiravir acts as a direct inhibitor of the viral RNA polymerase. As these agents exert their effects through distinct and non-overlapping mechanisms, an independence-based expectation provides the most appropriate null reference. The Bliss model captures this by estimating the combined antiviral effect as the probabilistic interaction of two mechanistically separate processes [44]. ZIP extends this approach by assessing whether either compound alters the apparent potency of the other across the response surface, a relevant consideration for host-targeting agents that can shift cellular susceptibility [45]. Both ZIP and Bliss showed lower scores but remained above the 10-point threshold for synergy. Surprisingly, we also observed a high, yet single-point, antagonistic effect between the two compounds at 1.25 µM of CH223191 and 2.5 µM of favipiravir.

The *in vivo* combination treatments also showed results suggesting an enhanced combined effect, as evidenced by the weight-loss protection afforded by the CH223191 (5 mg/kg/day) and sub-optimal favipiravir combination (Figure 5C). The beneficial interaction also translated into improved survival outcome (Figure 5A and Table 1). Additionally, viral titration of spleen samples showed a complementary decrease in TCRV titers in mice treated with the CH223191 (15 mg/kg/day) and sub-optimal favipiravir combination (Figure 6A). These results demonstrate that AHR antagonism synergizes with viral RNA polymerase inhibition *in vitro*, while showing a cooperative effect *in vivo* across distinct biological endpoints, depending on the AHR inhibitor concentration.

## 4. Materials and Methods

### 4.1. Animals

Male and female 20- to 22-day-old human transferrin receptor 1 (hTfR1) mice and 6- to 10-week-old AG129 interferon (IFN)-α/β and γ receptor-deficient mice were obtained from breeding colonies at Utah State University. The hTfR1 mice were fed Harlan Lab Block and tap water ad libitum. AG129 mice were fed irradiated Harlan Lab Block (Harlan Bioproducts for Science Inc, Indianapolis, IN, USA) and autoclaved tap water ad libitum.

### 4.2. Ethics Regulation of Laboratory Animals

All animal procedures used in this study complied with guidelines set by the United States Department of Agriculture and the Utah State University Institutional Animal Care and Use Committee.

### 4.3. Cell Cultures

Vero African green monkey kidney cells (ATCC^®^ CCL-81™, American Tissue Culture Collection, Manassas, VA, USA) were used for virus stock preparation and *in vitro* combination studies. Vero 76 cells (ATCC^®^ CRL-1587™) were also used for virus stock preparations.

### 4.4. Viral Strains

Tacaribe virus (TCRV), strain TRVL 11573, was obtained from the ATCC. The virus stock (2 passages in Vero 76 cells and 1 passage in AG129 mice) was prepared from clarified liver homogenates from AG129 mice challenged with TCRV. The virus stock was diluted in sterile Minimal Essential Medium (MEM) (Gibco, ThermoFisher Scientific, Waltham, MA, USA) for cell culture and mouse challenge studies. Vero cell cultures were infected at a MOI = 0.1 and AG129 mice were inoculated by intraperitoneal (IP) infection with 500 median cell culture infectious dose (CCID_50_) of TCRV in a 0.2 mL volume. A reverse genetics-derived Romero strain of JUNV was kindly provided by Dr. Slobodan Paessler (University of Texas Medical Branch) and has been previously described [46]. The stock used was prepared from a single passage in Vero cells and fully sequenced to confirm authenticity. The virus stock was diluted in sterile MEM, and the animals were challenged IP with a 0.2 mL volume containing approximately 10^4^ CCID_50_ of virus.

### 4.5. Compounds

CH223191 (2-(1H-indole-3-carbonyl)-thiazole-4-carboxylic acid methyl ester) was obtained from Sigma as a powder. Favipiravir (6-fluoro-3-hydroxy-2-pyrazinecarboxamide) was purchased from TargetMol. For the cell culture studies, both compounds were suspended in MEM containing 1.5% FBS at the final dosing concentrations of 20, 10, 5, 2.5, 1.25, 0.63, or 0.31 µM for CH223191 and 40, 20, 10, 5, 2.5, 1.25, or 0.63 µM for favipiravir. The favipiravir treatment for the hTfR1 mouse JUNV challenge study, was prepared in meglumine-based excipient. For the rest of the animal studies both compounds were suspended in sterile-filtered, 0.4% carboxymethyl cellulose (CMC) to a final concentration to deliver daily doses of 50, 15, 5, or 1.5 mg/kg for CH223191 and 200, 50, 16, 5, or 1.6 mg/kg for favipiravir.

### 4.6. Combined Antiviral Activity of CH223191 and Favipiravir in Cell Culture

Vero cells were plated in 96-well plates at a seeding density of 3 × 10^4^ cells/well. Cells were exposed to either a single drug or combinations of CH223191 and favipiravir for 3 h and incubated at 37 °C in a 5% CO_2_ atmosphere. The drugs were then removed and 50 µL of TCRV inoculum was added to each well and incubated for 1 h at 37 °C. After the infection, the viral inoculum was removed, and cells were exposed to fresh drug treatments. Following a 72 h incubation, cell supernatants were collected for viral yield determination by standard plaque assay. Inhibition percentages were calculated relative to viral controls. Additive/synergistic effects were analyzed using the Synergy Finder Plus 3.0 web application [47](Network Pharmacology for Precision Medicine group in the Research Program of System Oncology, Faculty of Medicine, University of Helsinki, Helsinki, Finland).

### 4.7. Animal Studies

Mice across all experiments were weighed 1 day before infection (day 0) and assigned to treatment groups so that sex and age were evenly distributed among groups. All experiments included a placebo group treated with the vehicle. Sham-infected, untreated mice were included as normal controls. To determine group sizes for the primary survival endpoint for the efficacy studies, we performed a power analysis using commonly accepted values for type I error (0.05) and power (80%). Formal randomization was not performed. Confounders were not controlled. Prior to the start of each experiment, mice underwent a 7-day acclimatization period within the ABSL-2 laboratory for TCRV studies and ABSL-3 for the JUNV studies. This duration allowed the animals to fully habituate to the vivarium environment and their specific housing cages. Room air temperature in the animal BSL-2 and BSL-3+ laboratories where the TCRV and JUNV studies were performed was 22.2  ±  4 °C with 30–70% air humidity. The rooms had a 12–12 h dark/light cycle. Any mice having lost 30% or more of their peak body weight were considered moribund and humanely euthanized per IACUC endpoint criteria protocol. All mice were anesthetized using 1% isoflurane in an anesthesia chamber prior to euthanasia. All animal procedures were conducted in strict accordance with the ethical standards and regulations established by the United States Department of Agriculture and the Animal Welfare Act. The study protocol was reviewed and approved by the Utah State University Institutional Animal Care and Use Committee. Approval Date: 30 August 2024 (for protocol 10034); 4 September 2024 (for protocol 10097).

Throughout the study, every effort was made to minimize animal suffering and to use the minimum number of animals necessary to achieve statistically valid results. No adverse effects were observed in any animal study.

In all animal studies, a single animal is considered an experimental unit. Laboratory technicians were blinded to treatments. Treatments were labeled in code to reduce risk of bias. Only lead researchers were aware of group allocations across all experimental stages. Primary outcome measure for all animal studies was survival.

#### 4.7.1. CH223191 Maximum Tolerated Dose Study

Mice (hTfR1) were administered 50, 15, 5, or 1.5 mg/kg CH223191 per os (PO) by oral gavage in a 0.1 mL volume. The compound was given once daily (q.d.) for 10 days. A group of untreated animals was included for baseline comparison. The mice were weighed daily and observed for 14 days for signs of toxicity and mortality. The design consisted of 6 groups: 50 (*n* = 10), 15 (*n* = 10) or 5 (*n* = 10) mg/kg CH223191, placebo (*n* = 9), 200 mg/kg favipiravir (*n* = 10), and sham infected (*n* = 4) with the total number of animals used being 53. A single placebo-treated mouse was excluded due to an early death on day 2 from a presumed oral gavage injury. Outcome measures evaluated were weight change and survival.

#### 4.7.2. CH223191 Efficacy Study in the hTfR1 JUNV Challenge Model

CH223191 (50, 15, or 5 mg/kg/day) was administered PO to hTfR1 mice beginning 2 h pre-infection and continued q.d. for 10 days. Favipiravir was administered by IP injection (0.1 mL) at 200 mg/kg following the same schedule. The animals were observed for 28 days for morbidity and mortality, and daily body weights were recorded. The design consisted of 6 groups: 50 (*n* = 6), 15 (*n* = 6), 5 (*n* = 6), or 1.5 (*n* = 6) mg/kg CH223191, placebo (*n* = 6) and sham infected (*n* = 4) with the total number of animals used being 34. No animals were excluded from this experiment. Outcome measures evaluated were weight change and survival.

#### 4.7.3. Favipiravir Sub-Optimal Dose Determination

Favipiravir (50, 16, 5, or 1.6 mg/kg/day) was administered PO (in 0.1 mL) to AG129 mice starting 2 h pre-infection and continued twice daily (b.i.d.) for a total of 10 days. The mice were observed for 21 days for morbidity and mortality. The design consisted of 6 groups: 50 (*n* = 8), 16 (*n* = 8), 5 (*n* = 8), or 1.6 (*n* = 8) mg/kg favipiravir, placebo (*n* = 8) and sham infected (*n* = 3) with the total number of animals used being 43. No animals were excluded from this experiment. Outcome measures evaluated were weight change and survival.

#### 4.7.4. CH223191 and Favipiravir Combination Study in the AG129 TCRV Challenge Model

A sub-optimal dose of favipiravir (30 mg/kg/day) was administered alone or in combination with 15 or 5 mg/kg/day of CH223191. Treatments were PO beginning 2 h pre-infection and continued b.i.d. for 10 days. The animals were observed for 28 days for morbidity and mortality, and daily body weights were recorded. Four preselected mice per group were sacrificed on day 9 post-infection (p.i.) to assess viremia and tissue viral titers. The design consisted of 8 groups: 5 mg/kg/day CH223191 and 30 mg/kg/day favipiravir (*n* = 10 *), 15 mg/kg/day CH223191 and 30 mg/kg/day favipiravir (*n* = 10), 5 mg/kg/day CH223191 (*n* = 10), and 15 mg/kg/day CH223191 (*n* = 10), favipiravir 30 mg/kg/day (*n* = 10), favipiravir 200 mg/kg/day (*n* = 6), placebo (*n* = 10 *) and sham infected (*n* = 3) with the total number of animals used being 69. Two animals were excluded from this experiment. The mouse from the 5 mg/kg/day CH223191 and 30 mg/kg/day favipiravir combination due to a dramatic early onset of weight loss was likely due to trauma associated with the IP inoculation. The mouse from the placebo group was removed from the study due to health concerns unrelated to the virus infection. Outcome measures evaluated were weight change, survival and tissue viral titers.

### 4.8. Spleen, Liver, and Serum Virus Titers

Blood samples were collected prior to euthanasia. For this purpose, blood was collected via submandibular venipuncture using 5 mm lancets. Following collection, blood samples were immediately placed on ice. Serum was subsequently isolated by centrifugation at 8000 RPM for 30 min at 4 °C. Spleen and liver samples were collected, weighed, and homogenized in MEM. Virus titers were assayed using an infectious cell culture assay as previously described [48]. Briefly, a specific volume of clarified tissue homogenate or serum was serially diluted and added to quadruplicate wells of Vero cell monolayers in 96-well microtiter plates. The viral cytopathic effect was determined 8 days after plating, and the 50% endpoints were calculated as described [49]. The lower detection limit was 2.27 log_10_ CCID50/g of tissue and 1.67 log_10_ CCID50/mL of serum. In samples where the virus was undetectable, a value representative of the lower detection limit was assigned for statistical analysis.

### 4.9. Statistical Analysis

The Mantel–Cox log-rank test was used to analyze Kaplan–Meier survival plots. Survival outcome was also analyzed using Fisher’s exact (two-tailed) test. An ordinary one-way ANOVA and a Dunnett post hoc test to correct for multiple comparisons were used to evaluate differences between the placebo and each experimental group for mean day-to-death (MDD), individual day weight change, and Tukey’s pos hoc test for percent inhibition of viral titers. All statistical evaluations were performed using Prism 10 (GraphPad Software, San Diego, CA, USA).

## 5. Conclusions

The results presented in this article further contribute to the growing evidence supporting AHR as a promising pharmacological target for the treatment of several viral infections. Notably, inhibition of the AHR with CH223191 effectively protected hTfR1 mice against a lethal JUNV infection. The protection was comparable to that achieved with the less-than-optimal q.d. dosing of favipiravir, a potent antiviral approved in Japan for the treatment of drug-resistant Influenza cases. Importantly, our findings also show that inhibition of the AHR, in combination with a sub-optimal dosing regimen of favipiravir, improves survival outcome, protects against weight loss, and decreases viral titers in organs targeted by TCRV infection in immunocompromised mice. The data support further exploration of adjunctive therapy with selective AHR antagonists in combination with antiviral drug candidates that directly target the virus replication cycle.

## Figures and Tables

**Table 1:** Summary of survival outcome and MDD for the CH223191 and favipiravir combination efficacy study in the TCRV AG129 mouse infection model.

Group	Infected Y or N	Test Articles & Doses	Survivors/Total ^a^ (MDD ^b^ ± SD)
1	Y	CH223191 5 mg/kg/day + Favipiravir 30 mg/kg/day	7/10 * (16 ± 2.0)
2	Y	CH223191 15 mg/kg/day + Favipiravir 30 mg/kg/day	4/10 (23 ± 3.8 ****)
3	Y	CH223191 5 mg/kg/day + 0.1 mL CMC	1/10 (16 ± 4.9 **)
4	Y	CH223191 15 mg/kg/day + 0.1 mL CMC	3/10 (11 ± 1.0)
5	Y	Favipiravir 30 mg/kg/day + 0.1 mL CMC	4/10 (16 ± 2.3 *)
6	Y	Favipiravir 200 mg/kg/day	6/6 **
7	Y	0.1 mL CMC + 0.1 mL CMC	1/9 (11 ± 1.5)
8	N	Sham-infected normal controls	3/3

^a^ Differences in the number of survivors between compound-treated and vehicle placebo-treated groups were analyzed by Fisher’s exact (two-tailed) test (* *p* < 0.05). ^b^ Mean day to death (MDD) of mice that succumbed during the infection was analyzed by ANOVA with Dunnett’s posttest. SD, standard deviation. **** *p* < 0.0001, ** *p* < 0.01 compared to the placebo-treated animals.

## Data Availability

The original contributions presented in this study are included in the article. Further inquiries can be directed to the corresponding author(s).

## Abbreviations

The following abbreviations are used in this manuscript:

AHR	Aryl Hydrocarbon Receptor
JUNV	Junín virus
TCRV	Tacaribe virus
PO	Per os
IP	Intraperitoneal
VHF	Viral hemorrhagic fever
q.d	Once daily
b.i.d	Twice daily
